# Supplementary material for: TRPV6 Channel Is Involved in Pancreatic Ductal Adenocarcinoma Aggressiveness and Resistance to Chemotherapeutics
Source: Cancers (Basel). 2023 Dec 8;15(24):5769. doi: 10.3390/cancers15245769 (PMC10741494; doi:10.3390/cancers15245769)
Supplement: Supplementary file 1 [file cancers-15-05769-s001.zip › cancers-2687099-supplementary.pdf]

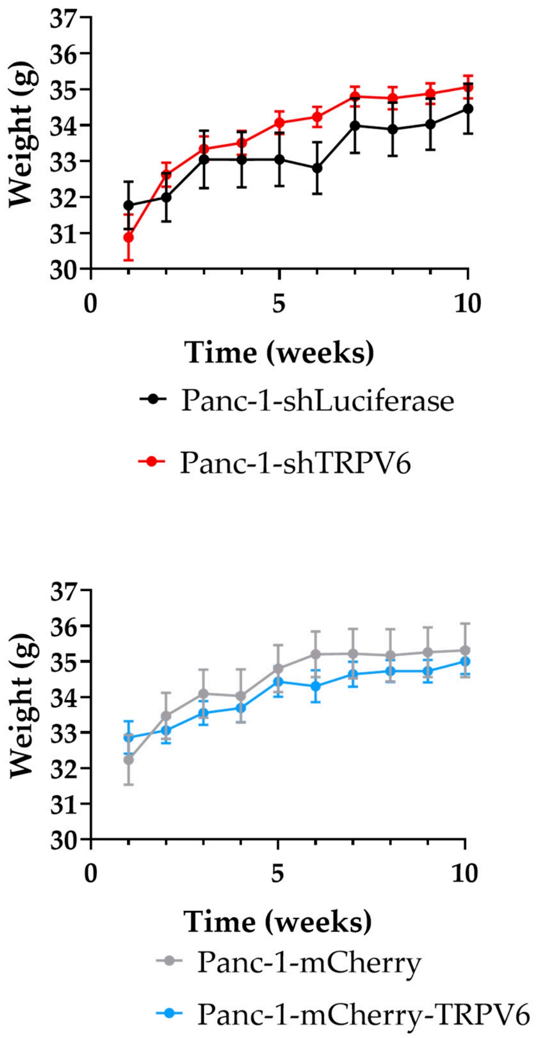

Figure S1. Weight curve of the animals throughout the in vivo experiment.

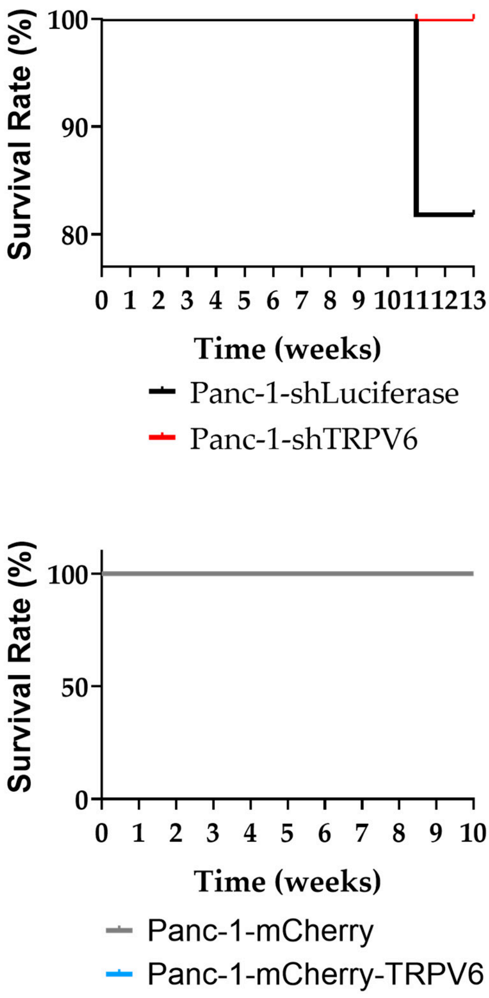

Figure S2. Survival curve of the animals throughout the in vivo experiment.
